# Evaluation of an Analogue of the Marine ε-PLL Peptide as a Ligand of G-quadruplex DNA Structures

**DOI:** 10.3390/md18010049

**Published:** 2020-01-11

**Authors:** Maria Marzano, Andrea Patrizia Falanga, Daniela Marasco, Nicola Borbone, Stefano D’Errico, Gennaro Piccialli, Giovanni Nicola Roviello, Giorgia Oliviero

**Affiliations:** 1 Department of Pharmacy, University of Naples Federico II, Via Domenico Montesano 49, 80131 Naples, Italy; 2 Institute of Biostructures and Bioimaging—CNR 1, Via Mezzocannone 16, 80134 Naples, Italy; 3 Department of Molecular Medicine and Medical Biotechnologies, University of Napoli Federico II, Via Sergio Pansini 5, 80131 Naples, Italy

**Keywords:** marine peptide, epsilon-poly-l-lysine, ε-PLL, G-quadruplex DNA, human telomere, c-myc oncogene

## Abstract

ε-poly-l-Lysine (ε-PLL) peptide is a product of the marine bacterium *Bacillus subtilis* with antibacterial and anticancer activity largely used worldwide as a food preservative. ε-PLL and its synthetic analogue α,ε-poly-l-lysine (α,ε-PLL) are also employed in the biomedical field as enhancers of anticancer drugs and for drug and gene delivery applications. Recently, several studies reported the interaction between these non-canonical peptides and DNA targets. Among the most important DNA targets are the DNA secondary structures known as G-quadruplexes (G4s) which play relevant roles in many biological processes and disease-related mechanisms. The search for novel ligands capable of interfering with G4-driven biological processes elicits growing attention in the screening of new classes of G4 binders. In this context, we have here investigated the potential of α,ε-PLL as a G4 ligand. In particular, the effects of the incubation of two different models of G4 DNA, i.e., the parallel G4 formed by the Pu22 (d[TGAGGGTGGGTAGGGTGGGTAA]) sequence, a mutated and shorter analogue of the G4-forming sequence known as Pu27 located in the promoter of the c-myc oncogene, and the hybrid parallel/antiparallel G4 formed by the human Tel22 (d[AGGGTTAGGGTTAGGGTTAGGG]) telomeric sequence, with α,ε-PLL are discussed in the light of circular dichroism (CD), UV, fluorescence, size exclusion chromatography (SEC), and surface plasmon resonance (SPR) evidence. Even though the SPR results indicated that α,ε-PLL is capable of binding with µM affinity to both the G4 models, spectroscopic and SEC investigations disclosed significant differences in the structural properties of the resulting α,ε-PLL/G4 complexes which support the use of α,ε-PLL as a G4 ligand capable of discriminating among different G4 topologies.

## 1. Introduction

**Figure 1:**
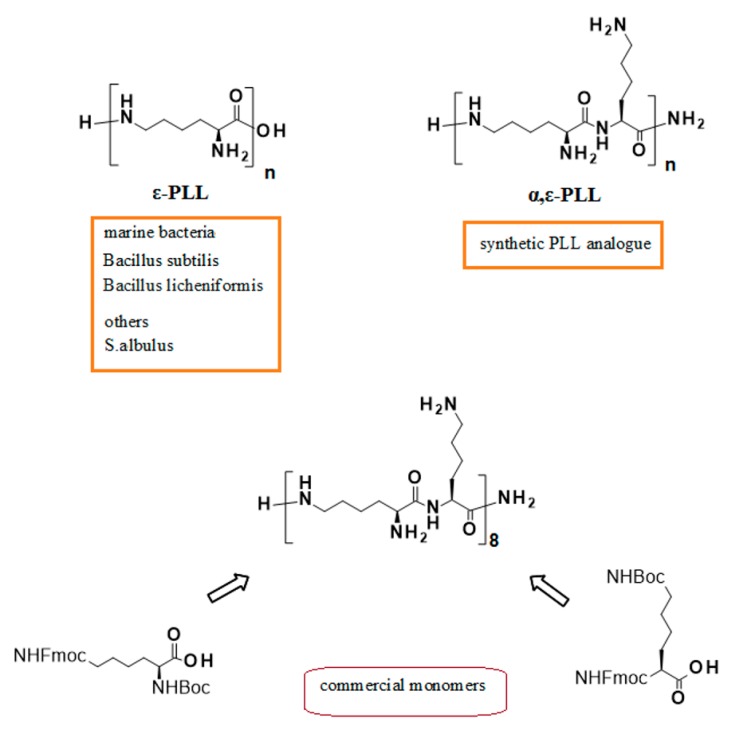
Schematic representation of the natural ε-peptide (ε-PLL) and of our synthetic analogue studied in G-quadruplex (G4)-DNA binding.

ε-poly-l-Lysine (ε-PLL) is a cationic biopolymer isolated from the marine bacterium *Bacillus subtilis,* responsible for the antibacterial and anticancer activity shown by this microorganism [1]. The same peptide is also produced by the marine bacterial strain PL26 of *Bacillus licheniformis*, isolated from the west coast of India [2]. Due to its well-known antimicrobial properties, ε-PLL is largely used worldwide as a food preservative [3,4], but is also used in many biomedical applications, including the enhancement of some anticancer agents [5], the suppression of the production of the prion protein in neurodegenerative disorders [6], the use in contrast agent probes for Magnetic Resonance Imaging [7] and the enhancement of gene delivery efficiency [8]. The industrial production of ε-PLL makes use of a mutant of *Streptomyces albulus* [9]. Still, it can be conveniently achieved also using the above mentioned strain PL26 of marine bacterium *Bacillus licheniformis* starting from waste material from biodiesel manufacturing industries [1,2]. On the other side, α-poly-l-lysine (α-PLL) is a synthetic poly(amino acid) successfully used in different biotechnological applications, e.g., in biomass production by microalgae *Chlorella ellipsoidea* [10]. α- and ε-PLL peptides are well soluble in aqueous media, biodegradable, and environmental-friendly [11], and both are good candidates as drug delivery agents due to their polycationic nature [11,12]. Even though dendrimeric α,ε-poly-l-lysines had been previously realized for the compacting and delivery of oligonucleotides [13], a synthetic approach to linear PLLs with sequential α- and ε-peptide bonds (α,ε-PLLs, Figure 1) was firstly reported by Roviello et al. alongside with the initial biological assessment of α,ε-PLLs [13,14,15]. The interest in poly-l-lysine structures containing both α- and ε- peptide bonds is justified by their superior gene delivery properties when compared to linear or dendritic PLLs based uniquely on ε-peptide bonds [16]. PLLs having a random α- and ε- peptide bond sequence (hyperbranched polylysines) are more resistant to proteolytic action than linear PLL, but undergo a significant degradation after 8 h [17]. On the other side, linear α,ε-PLLs, easily obtainable by standard solid-phase peptide synthesis procedures [15,18,19] and endowed with structural specific nucleic acids binding abilities, do not show any significant degradation after 24 h of incubation in human serum at 37 °C [14].

Among the DNA secondary structures, the G-quadruplex (G4) family is one of the most intriguing and deeply investigated [20,21,22,23]. It has been demonstrated that G4 DNA plays a crucial role in many physiological and disease-related biological mechanisms [24]. Apart from the ubiquitous potassium or sodium cations, positively-charged polyamines and triethylene tetraamine may contribute to the G4 stability and induce biologically-relevant effects [25,26]. In this context, also the polycationic PLL was evaluated for its impact on the formation of G4 structures by the human telomere in cation-deficient media and showed the interesting ability to convert the telomeric G4 from the antiparallel to the parallel topology [27]. However, to our knowledge, no study has yet been published on the interaction of α,ε-PLL with any G4 DNA.

Thus, herein, we report the results of our study on the effects of α,ε-PLL on two different G4 topologies investigated by CD, UV, fluorescence, size exclusion chromatography and SPR techniques. The hybrid-type G4 adopted by the Tel22 telomeric sequence (d[AGGGTTAGGGTTAGGGTTAGGG]), commonly used as the human telomeric DNA model, and the parallel G4 formed by the Pu22 (d[TGAGGGTGGGTAGGGTGGGTAA]) sequence, a shorter and mutated analogue of the G4-forming Pu27 sequence located in the promoter of the human c-myc oncogene, have been employed for this study [28,29]. These two model DNAs can adopt several kinds of topologies under different experimental conditions and are used in this study to evaluate the ability of our lysine-rich peptide to modify, similarly to other oligocation binders [27,30,31], G4 DNA structures.

The results of this study have suggested that α,ε-PLL is a G4 ligand able to bind to both G4 models with µM affinity leading, however, to more evident changes in the secondary structure of parallel G4 structures as described hereinbelow.

## 2. Results and Discussion

**Figure 2:**
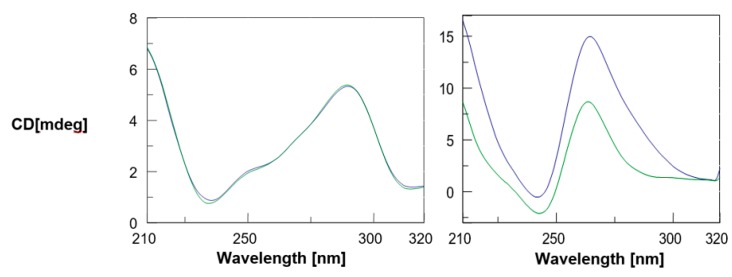
CD spectra of 2.5 µM G4 DNA (Tel22, **left**; Pu22, **right**) + 1.7 µM α,ε-PLL in 10 mM TRIS HCl buffer, 100 mM KCl, pH 7.4 at 15 °C. Arithmetic sum (▬); α,ε-PLL/G4 complex (▬).

The interest towards the biomedical exploitation of DNA G-quadruplexes and their ligands (of either natural or synthetic origin) prompted us to explore the interaction of α,ε-PLL with two different structural topologies of this class of highly-ordered secondary structures of DNA. In all the spectroscopic studies described below, the contribution to the spectra given by the free peptide was negligible when compared to the intense nucleic acid bands, as also previously reported by Roviello et al. [14]. At first, we studied the interaction of α,ε-PLL with Tel22, that under our experimental conditions showed the characteristic CD bands (~290 nm maximum, ~250–260 nm shoulder, ~240 nm minimum) previously attributed to an equimolar mixture of the hybrid 1 and hybrid 2 G4s [32,33,34,35]. Following the addition of α,ε-PLL at the $\frac{1}{2}\frac{\left[{\mathrm{NH}}_{3}^{+}\right]}{\left[{\mathrm{PO}}_{3}^{–}\right]}$ ratio (at which ε-PLL caused the largest structural perturbation of telomeric DNA [27]) we observed no change in the CD spectrum of Tel22, reaching the conclusion that α,ε-PLL did not induce any significant structural change on the G4 secondary structure of Tel22 (Figure 2, left). Afterwards, the same study was repeated using the c-myc-derived Pu22 DNA sequence which is known to fold into a parallel G4 as previously demonstrated by NMR [36,37] and X-ray crystallography [38], and confirmed in our study by the CD profile dominated by the negative Cotton effect centered at around 240 nm and the positive one centered at about 265 nm (Figure 2, right) which are typically assigned to parallel G4s [39,40]. Immediately after the addition of α,ε-PLL, we observed a substantial enhancement and broadening of the positive CD band at 260 nm, which indicated that the interaction of α,ε-PLL with Pu22 induced significant changes into the parallel G4 secondary structure. Interestingly, no significant change in CD spectrum of a dsDNA upon peptide addition was detected under the same experimental conditions adopted for G4 DNAs (Figure S1).

**Figure 3:**
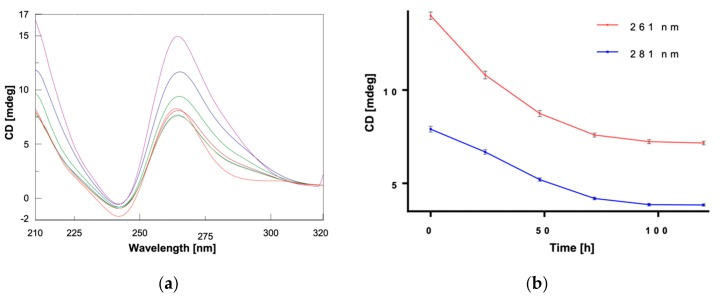
(**a**) CD spectra of Pu22 (2.5 µM) recorded before (▬) and 0 (▬), 24 (▬), 48 (▬), 72 (▬), 96 (▬), and 120 (▬) h after mixing with α,ε-PLL (1.7 µM) in 10 mM TRIS HCl buffer, 100 mM KCl, pH 7.4 at 15 °C. (**b**) Plots of CD values vs. time at 261 and 281 nm.

**Figure 4:**
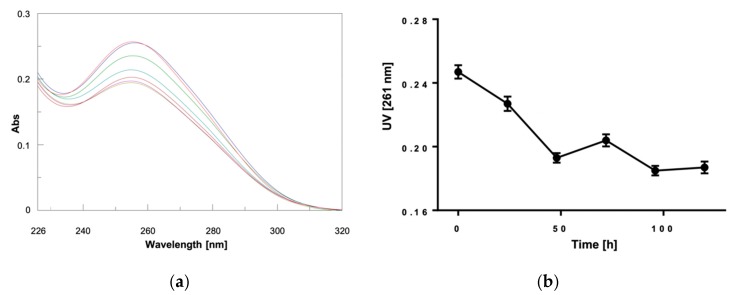
(**a**) UV spectra relative to Pu22 (2.5 µM) in 10 mM TRIS HCl buffer, 100 mM KCl, pH 7.4, b = 0.4375 cm, at 15 °C recorded before (▬) and 0 (▬), 24 (▬), 48 (▬), 72 (▬), 96 (▬), and 120 (▬) h after mixing with α,ε-PLL (1.7 µM); (**b**) Plot of the UV values at 261 nm vs. time.

By repeating the acquisition of CD (Figure 3) and UV (Figure 4) spectra at different time points from the addition of α,ε-PLL to the preformed Pu22 G4, we observed a continuous variation of the signals (Figure 3a and Figure 4a) which was particularly evident by plotting the CD value recorded at 261 or 281 (Figure 3b) nm, as well as the UV value at 261 nm (Figure 4b) vs. time.

From this kinetic study, we concluded that the interaction of α,ε-PLL with the Pu22 G4 leads to the formation of complexes that evolve during the first 96 h after mixing and eventually reach the most stable one when the stabilization of CD signal was achieved (Figure 3). Interestingly, the UV titration experiment clearly showed a hypochromic effect (Figure 4, Figure S2) occurring upon peptide addition to Pu22 that suggests a reinforcement of the stacking between the G-tetrads as an outcome of the binding with the α,ε-PLL.

All the time-dependent spectroscopic changes observed in the above-reported experiments are due to the binding with peptide ligand, as Pu22 G4 alone did not undergo a similar kinetic behavior in the time range explored (data not shown).

**Figure 5:**
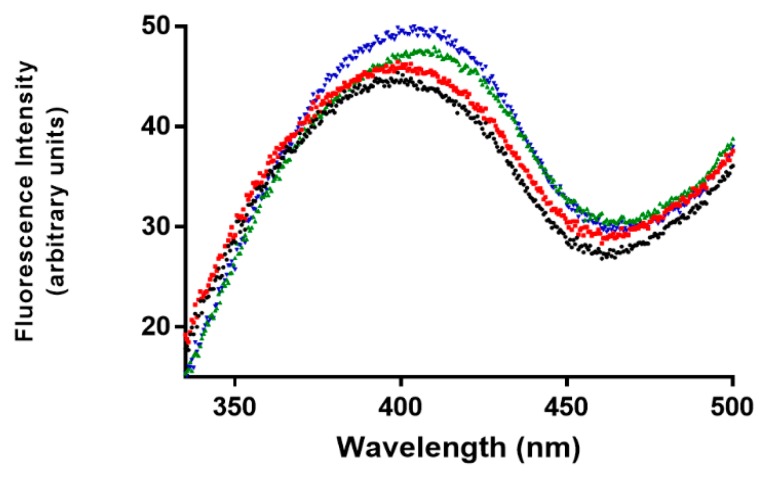
Fluorescence emission spectra of Pu22 (**▬**), Tel22 (**▬**), Pu22 + α,ε-PLL (**▬**) and Tel22 + α,ε-PLL (**▬**): 2.5 µM DNA, α,ε-PLL (1.7 µM) in 10 mM TRIS HCl buffer, 100 mM KCl, pH 7.4 (15 °C).

Similarly to CD, also fluorescence spectra confirmed a higher structural perturbation for Pu22 G4 DNA with respect to the telomeric one after binding with α,ε-PLL. We observed a slight blue-shift effect associated with increased fluorescence emission in the former case, whereas we did not see any significant effect in the latter case (Figure 5). We also investigated the effects induced by α,ε-PLL on the apparent melting temperatures of the two G4 models.

**Figure 6:**
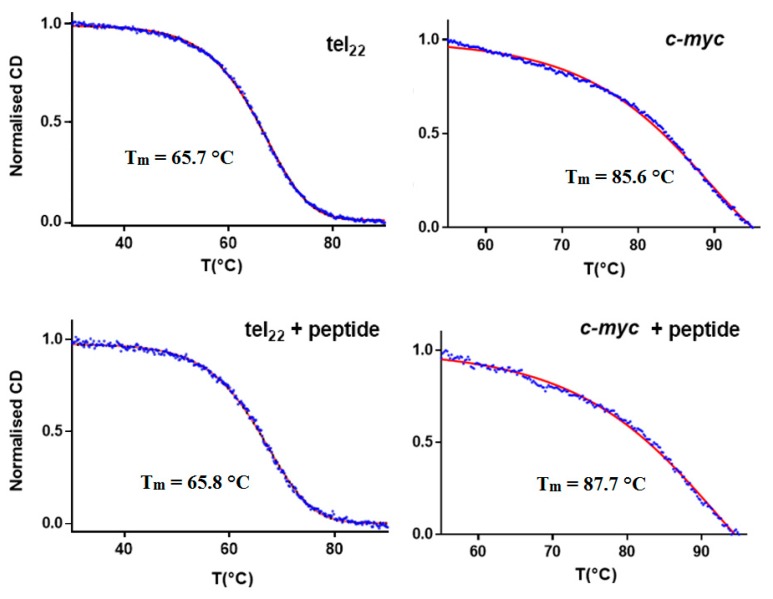
CD melting curves relative to G4 DNA (tel22, **top left**; *c-myc,* **top right**; 2.5 µM) and their complexes with 1.7 µM α,ε-PLL (tel22 + α,ε-PLL, **bottom left**; *c-myc* + α,ε-PLL, **bottom right**) in 10 mM TRIS HCl buffer, 100 mM KCl, pH 7.4.

The CD melting curves of the α,ε-PLL/G4 complexes (Figure 6 and Table 1) disclosed a slight thermal stabilization (ΔT_m_ ~2 °C) in the case of Pu22, whereas no significant difference was found between the T_m_ value of Tel22 and that of its complex with α,ε-PLL.

**Figure 7:**
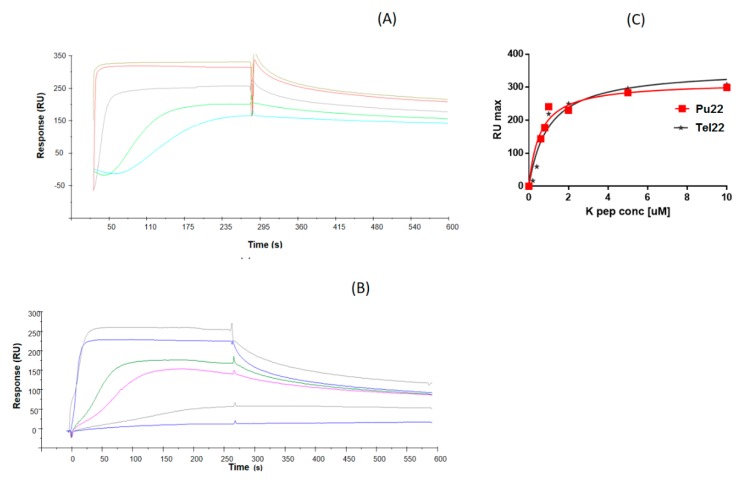
Overlay of surface plasmon resonance (SPR) sensorgrams for the binding of α,ε-PLL to immobilized (**A**) Biot-Pu22 or (**B**) Biot-Tel22. (**C**) Overlay of the corresponding binding isotherms of RU_max_ values vs. α,ε-PLL concentration (0–10 µM concentration range).

Overall, the above findings suggest the ability of α,ε-PLL to perturb parallel G4 structures leaving almost unchanged hybrid ones as testified by the spectral consequences seen only for Pu22, whose G4 structure also underwent a slight thermal stabilization. Even though the spectroscopic analyses indicated that the α,ε-PLL peptide is provided with a certain degree of selectivity in G4 structural modification, we decided to ascertain whether it was capable of binding also the Tel22 G4, as we expected on the basis of the electrostatic interactions foreseen for a polycation (α,ε-PLL) interacting with a polyanionic DNA. To this scope, and to quantitatively evaluate the binding affinity of α,ε-PLL for both the studied G4 topologies, we used the surface plasmon resonance (SPR) technique that had proven successful in other literature examples [41]. SPR binding profiles (Figure 7) confirmed that α,ε-PLL was actually capable of binding, in a concentration-depending manner, to both the G4 models, and the fitting of the binding isotherms allowed us to estimate the dissociation constants for the two complexes, which were quite comparable even though a higher affinity for Pu22 was observed (see Table 2), in the interval time of analysis. Strikingly, the affinity of α,ε-PLL for Pu22 and Tel22 G4 DNA is similar or higher than that found for other previously reported basic peptides acting as G4 DNA ligands (K_D_ 1–8 µM) [42].

Overall, the interaction of α,ε-PLL with Pu22 G4 DNA was unambiguously proven by SPR, CD, UV and fluorescence spectroscopies, while Tel22 binding was evidenced only by SPR that revealed, however, a binding affinity for Tel22 (and for Pu22) similar when not higher than that typically found for peptide G4 DNA ligands [42]. It was previously proven that, due to its polycationic nature, α,ε-PLL was able to bind several nucleic targets [14,15] inducing, however, structural rearrangements in its nucleic targets that varied upon their different nature (RNA vs. DNA) and nucleobase sequence [14,15]. This suggested, thus, that other interaction forces besides the obvious ionic ones may determine the overall binding process. In this context, we here paid great attention to the comparison between the effects of α,ε-PLL on a parallel (Pu22) and a hybrid (Tel22) quadruplex structure. More in detail, as shown by CD and fluorescence experiments, only Pu22 underwent a significant secondary structure rearrangement upon peptide binding, an aspect that was explored also in a kinetic study. Nevertheless, the expected electrostatic interactions led α,ε-PLL to bind also Tel22 (as found by SPR) without determining, however, any CD spectral changes in this case and, correspondingly, leaving almost unmodified its secondary structure. Other differences found in the two cases comprise the higher binding affinity of the peptide for Pu22 with respect to Tel22, as evidenced by SPR comparing the K_D_ values for the two complexes (Table 2, Figure 7), and the slight thermal stabilization of Pu22 (but not of Tel22) found by a CD-melting assay (Table 1).

**Figure 8:**
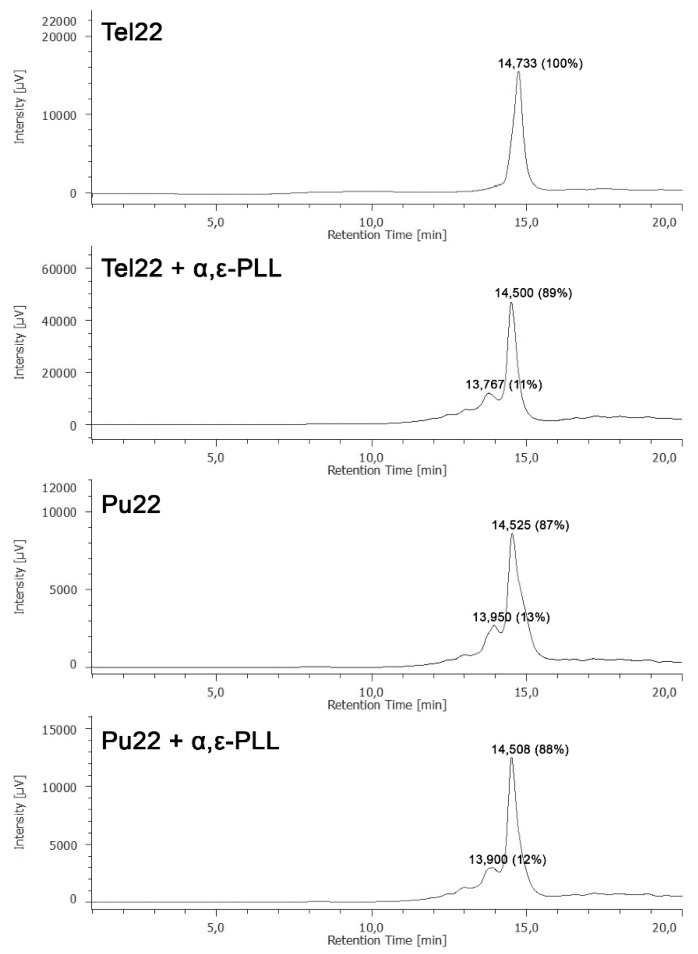
HPLC-size exclusion chromatography (SEC) profiles of Tel22 and Pu22 G4s before and after the addition of α,ε-PLL.

Aiming at further confirming the binding of α,ε-PLL with the two G4 models and also at investigating the effect of the addition of α,ε-PLL on the molecularity of Tel22 and Pu22 G4s, we used the HPLC size exclusion chromatography (HPLC-SEC) technique. In other papers, in fact, we and others demonstrated that the HPLC-SEC could be successfully exploited to assess the molecularity and conformational changes of DNA G4s [43,44,45]. Accordingly, we recorded the HPLC-SEC profiles of Tel22 and Pu22 G4s before and 96 h after their incubation with α,ε-PLL (Figure 8). As expected, the HPLC-SEC profile of Tel22 alone was populated by a single peak attributable to the mixture of monomeric hybrid 1 and hybrid 2 G4s, which was eluted at min 14.733. After incubation with α,ε-PLL, the appearance of a new less-retained peak eluted at min 13.767 suggested the formation of a higher molecular weight complex in agreement with the SPR data which indicated a concentration-dependent binding between α,ε-PLL and Tel22. Differently from what was observed for Tel22 G4, the HPLC-SEC profile obtained by injecting Pu22 alone was populated by two main peaks, that we assigned to the monomeric parallel G4 (*t*_R_ = 14.525 min) and its dimer (*t*_R_ = 13.950 min) based on literature reports [38]. On the other hand, the HPLC-SEC profile of Pu22 did not change significantly after the addition of α,ε-PLL, suggesting that the α,ε-PLL/Pu22 binding confirmed by CD, fluorescence and SPR data, did not affect the relative abundance of the monomeric and dimeric G4 forms.

## 3. Materials and Methods

### 3.1. α,ε-PLL and DNA

The hexadecapeptide α,ε-PLL (Figure 1) was prepared accordingly to the procedure previously reported by Moccia et al. [15]. Pu22 (d[TGAGGGTGGGTAGGGTGGGTAA]) and Tel22 (d[AGGGTTAGGGTTAGGGTTAGGG]) G4-forming DNAs were purchased from Eurofins Genomics.

### 3.2. CD and UV Experiments

We obtained the CD spectra in a 210–320 nm wavelength range, on a Jasco J-810 spectropolarimeter equipped with a Jasco PTC-423S/15 Peltier temperature controller (Jasco Europe Srl, Cremella, Italy) in a dual-chamber quartz cell (b = 2 × 0.4375, Hellma 238-QS, Hellma^®^ Analytics, Hellma GmbH & Co. KG, Müllheim, Germany). UV spectra were recorded on a Jasco V-550 spectrophotometer equipped with a Jasco ETC-505T Peltier temperature controller. All spectroscopic experiments were run in duplicate and were repeated three times. Standard deviation values for CD and UV Abs were ≤2%. CD meltings of telomeric and Pu22 G4 DNAs were obtained by recording CD values at 295 and 263 nm, respectively, as function of temperature. All nucleic acids were annealed by heating the solutions at 95 °C for 5 min and letting them cool overnight at room temperature. Melting temperature values were determined as the temperatures relative to minima of the 1st derivative plots of denaturation curves.

### 3.3. Fluorescence Studies

The experiments were performed at 15 °C using the intrinsic fluorescence of G4 DNAs as reported previously [46], using an excitation wavelength of 270 nm and a fluorescence emission wavelength ranging from 330 to 500 nm on a Jasco FP 8300 spectrofluorometer using a 10 mm path-length quartz cuvette. The acquisition parameters were set as follows: excitation and emission slits at 5 nm; 200 nm/min scan rate; 0.5 nm data interval averaging time at 0.050 s, PMT voltage at “medium”.

### 3.4. Surface Plasmon Resonance (SPR) Experiments

SPR assays were performed at 25 °C on a Biacore 3000 instrument (GE Healthcare, Chicago, IL, US). For immobilization, 5’-biotinylated Tel22 and Pu22 ODNs were injected for 7 min (at 20 µM) on a SA-chip until an immobilization of ~700 RU was achieved, as previously reported [47,48]. The α,ε-PLL analyte was serially diluted in the 10 µM TRIS HCl buffer supplemented with 100 µM KCl (pH 7.4) running buffer, covering a concentration range between 50 and 1000 nM. 90 µL analyte samples were injected at a flow rate of 20 µL/min and the dissociation was followed for 300 s. The reference chip sensorgrams were properly subtracted to sample sensorgrams. After each cycle, the sensor chip surface was regenerated with a 1.0 M NaCl solution for 30 s followed by multiple buffer injections to yield a stable baseline for the following cycles. Experiments were carried out in duplicates. Kinetic parameters were estimated assuming a 1:1 binding model and using version 4.1 Evaluation Software (GE Healthcare).

### 3.5. HPLC-Size Exclusion Chromatography (SEC) Analyses

HPLC-SEC was performed using a Phenomenex (Bologna, Italy) Yarra SEC-2000 column (300 × 7.8 mm, 3 µm) eluted with 90 mM KCl and 10 mM KH_2_PO_4_/CH_3_CN (80:20, *v*/*v*), flow rate 0.6 mL/min, UV-detector at 260 nm. The analyses were performed at room temperature.

## 4. Conclusions

Overall, the above-reported findings showed that α,ε-PLL binds the two herein investigated DNA G-quadruplexes with good affinity (as demonstrated by SPR), though clues of changes of the secondary structure and slight thermal stabilization were seen only for the interaction with the Pu22 parallel G4. Following complexation with α,ε-PLL, Pu22 G4 underwent significant alterations of its spectroscopic (CD, UV and fluorescence) properties. In particular, the slight blue-shift in the fluorescence band of peptide/Pu22 complex and the hypochromic UV effect indicated that the interaction with α,ε-PLL increased base stacking within the G4 core. In conclusion, the here presented study provides an interesting insight into G-quadruplex binding of α,ε-PLL, a serum stable [14], marine-inspired peptide that, in spite of its cationic nature, recognizes the structural properties of its targets and uses different binding modes according to their topology.

## Figures and Tables

**Table 1:** Melting temperatures from CD thermal denaturation experiments.

	Tm [°C]	Error	ΔTm [°C]
Tel22	65.7	0.2	–
Tel22 + α,ε-PLL	65.8	0.2	0.1
Pu22	85.6	0.2	–
Pu22 + α,ε-PLL	87.7	0.2	2.1

**Table 2:** SPR-based equilibrium dissociation constants (K_D_) for the interaction of α,ε-PLL with Pu22 or Tel22 G-quadruplexes. The Graph-Pad Prism software (version 7.00; GraphPad Software, San Diego, CA, USA) was used to fit RUmax for α,ε-PLL concentrations by nonlinear regression analysis.

ODN	K_D_ (µM)
Pu22	0.56 ± 0.14
Tel22	1.0 ± 0.20

## Supplementary Materials

Additional CD and UV data are available online at https://www.mdpi.com/1660-3397/18/1/49/s1.
