# Supplementary material for: Evaluation of an Analogue of the Marine ε-PLL Peptide as a Ligand of G-quadruplex DNA Structures
Source: Mar Drugs. 2020 Jan 11;18(1):49. doi: 10.3390/md18010049 (PMC7024349; doi:10.3390/md18010049)
Supplement: Supplementary file 1 [file marinedrugs-18-00049-s001.pdf]

Supporting information

Evaluation of an analogue of the marine ε-PLL peptide as a ligand of G-quadruplex DNA structures

M. Marzano[a], A. P. Falanga[a], D. Marasco[a], N. Borbone[a], S.D'Errico[a], G. Piccialli[a], G. N. Roviello[b,*], and G. Oliviero[c]

[a]Department of Pharmacy, University of Naples Federico II, Via Domenico Montesano 49 - 80131, Naples

[b]Institute of Biostructures and Bioimaging -CNR 1, Via Mezzocannone 16 - 80134, Naples

[c]Department of Molecular Medicine and Medical Biotechnologies, University of Napoli Federico II, Via Sergio Pansini 5 - 80131, Naples

*correspondence to G. N. Roviello, e-mails: giovanni.roviello@cnr.it; giroviel@unina.it

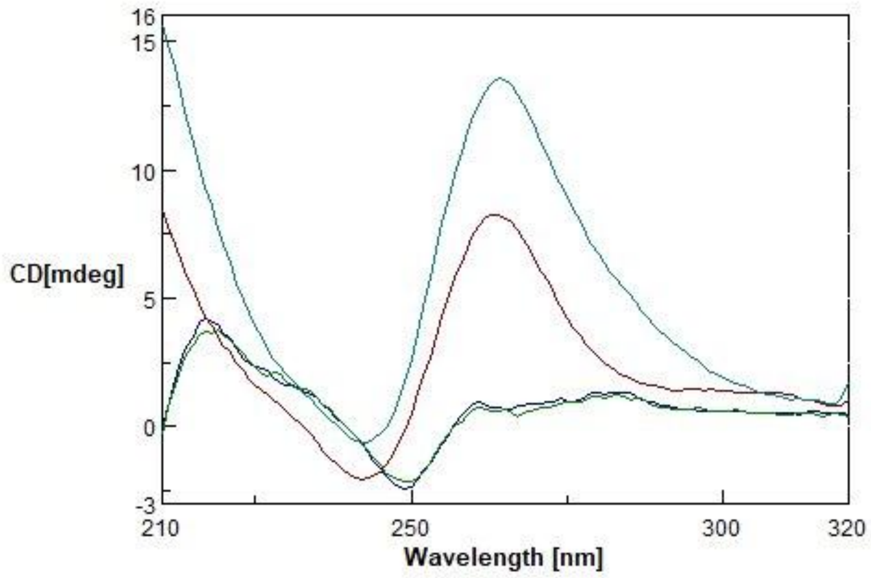

Figure S1. Sum and complex CD spectra of 2.5 μM DNA+alpha,epsilon-PLL before (dA12/dT12 - green, Pu22 – red) and immediately after (dA_{12}/dT_{12} – blu, Pu22 – azure) peptide addition to DNA solutions in 10 mM TRIS HCl buffer, 100 mM KCl, pH 7.4 at 15 °C.

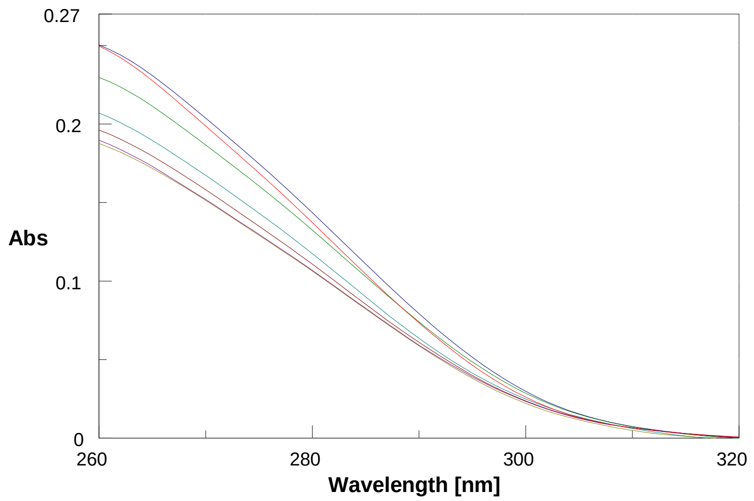

Fig S2. Expansion of the UV spectra relative to Pu22 (2.5 μM) in 10 mM TRIS HCl buffer, 100 mM KCl, pH 7.4 at 15 ℃ recorded before (—) and 0 (—), 24 (—), 48(—), 72(—), 96 (—), and 120 (—) h after mixing with α,ε-PLL (1.7 μM) in the 260-320 nm range.
